# Young women's engagement with gambling: A critical qualitative inquiry of risk conceptualisations and motivations to gamble

**DOI:** 10.1002/hpja.651

**Published:** 2022-08-24

**Authors:** Simone McCarthy, Samantha Thomas, Hannah Pitt, Sarah Marko, Melanie Randle, Sean Cowlishaw, Sylvia Kairouz, Mike Daube

**Affiliations:** ^1^ Institute for Health Transformation, Faculty of Health Deakin University Geelong Vic. Australia; ^2^ Institute for Health Transformation Deakin University Geelong Vic. Australia; ^3^ Faculty of Business and Law University of Wollongong Wollongong NSW Australia; ^4^ Department of Psychiatry The University of Melbourne Melbourne Vic. Australia; ^5^ Department of Sociology and Anthropology Concordia University Montreal QC Canada; ^6^ Faculty of Health Sciences Curtin University Perth WA Australia

**Keywords:** evidence‐based practice, gambling, health advocacy, qualitative methods, social determinants, women's health

## Abstract

**Background:**

Younger women's engagement with gambling has changed over recent decades due to a range of socio‐cultural, environmental and commercial factors. However, younger women's distinct lived experiences with gambling have rarely been considered. The following critical qualitative inquiry explored factors that influenced younger women's engagement with gambling and their perceptions of gambling risks.

**Methods:**

Semi‐structured interviews were conducted with 41 Australian women aged 18‐40 years. Participants were asked questions relating to their reasons for gambling, and the perceived risks associated with gambling. Reflexive thematic analysis was used to interpret the data.

**Results:**

Five themes were constructed from the data. First, women reported that they gambled to escape their everyday lives, with some women reporting gambling within their own homes. Second, women reported gambling for financial reasons, particularly to change their life circumstances and outcomes. Third, gambling was used by women as a way to connect with social network members. Fourth, gambling was an incidental activity that was an extension of non‐gambling leisure activities. Finally, lower risk perceptions of participants' own gambling risk contributed to their engagement and continuation of gambling.

**Conclusion:**

Public health and health promotion initiatives should recognise that young women's gambling practices are diverse, and address the full range of socio‐cultural, environmental and commercial factors that may influence younger women's engagement with gambling.

## 1 INTRODUCTION

Gambling is a significant global public health issue, and an important target for harm prevention strategies and public health interventions. Despite women participating in gambling at similar rates to men, and a significant proportion of the female population at risk of gambling‐related harm,^2^, ^3^, ^4^ women are rarely considered a priority group for gambling harm prevention activities or research.^5^ The disproportionate focus on the gambling behaviours of men has contributed to unintended consequences for women who gamble, including intensifying stigma and perpetuating stereotypes that may make it difficult for women to recognise gambling‐related risks and seek help.^5^ Researchers have thus proposed a gendered approach to gambling harm prevention, in order to ensure that women's unique experiences and needs are considered in gambling research, policy and prevention activities.^6^, ^7^

A small but increasing number of studies in public health and the social sciences have started to investigate women's gambling experiences. Researchers have documented a range of individual, socio‐cultural, environmental and commercial (or gambling industry related) factors that may contribute to women's engagement with gambling products and environments.^7^, ^8^, ^9^ These researchers have also explored women's perceptions of the risks associated with gambling,^10^ and their experiences of gambling‐related harm.^11^, ^12^ Relevant studies have identified women's unique experiences and social practices associated with gambling (as compared to men), and also report distinctive appeal factors associated with gambling for women.^13^, ^14^

Women's conceptualisations of gambling risk may be embedded in their unique socio‐cultural contexts, including constructs of motherhood, accountability for household essentials and providing for children.^15^, ^16^ For migrant women in particular, gambling motivations and conceptualisation of risk may centre around their role within domestic households, whereby gambling may function as an escape from systemic disadvantage associated with gender, race and class.^17^ Traditional gender constructs may also increase women's vulnerability to gambling‐related harm, while pressures to uphold normative social expectations of gender roles may prevent them from seeking help.^18^ In part, this may be because women have faced significant stigma for participating in historically male dominated gambling activities, and thus experience shame for seeking help for gambling harm.^19^

Previous research from Australia has demonstrated that younger women aged 18‐24 years are the most at‐risk subgroup of women for gambling‐related harm, and have the highest rates of moderate risk and problem gambling as compared to other age groups.^20^ Relative to middle‐aged (35‐54 years) and older women (55 years and over), cross‐sectional analyses suggest that younger women (16‐34 years) gamble more frequently, are more likely to experience severe levels of gambling harm, gamble on multiple products, and have a lower perception of harms associated with some forms of gambling.^21^ While researchers have demonstrated that young men are increasingly engaged in online gambling,^4^, ^20^ they have also suggested that younger women's engagement with gambling is changing over time.^4^, ^21^ For example, Thomas and colleagues ^22^ identified that online forms of gambling, such as sports betting, may appeal to and influence younger women's gambling behaviours, while increases in the socio‐cultural acceptance of gambling may contribute to younger women's reduced perceptions of risks associated with gambling. Such findings raise important questions about how different groups of women may conceptualise the risks associated with gambling. How individuals conceive risk behaviours is arguably a product of many social practices and dynamics, and is shaped by social contexts, social experiences, and imagined futures.^23^, ^24^ As Zinn^24^ ^(p1)^ has argued, perceptions of risk are complex, at times contradictory and shaped by structural and cultural factors, and can be central to how individuals develop and protect valued identities. However, there has been very limited research exploring how these factors may shape younger women's conceptualisation of the risks associated with their gambling, and the impact of this on their engagement with different gambling products.

This study aimed to explore factors that may influence younger women's motivations to gamble, and their perceptions of the risks associated with gambling. Three research questions guided the study:

1. What are the range of factors that influence younger women's reasons for gambling?
2. How do younger women conceptualise the risks associated with gambling?
3. How can public health responses address younger women's initiation, continuation and perceptions of the risks associated with gambling?

## 2 METHODS

### 2.1 Approach

This research was part of a larger study investigating the normalisation of gambling for younger women in Australia. One other paper from this dataset has explored women's perceptions of strategies to address the normalisation of gambling.^25^ The current study adopted a public health perspective and a critical qualitative approach to inquiry.^26^ The public health approach considers the broader impact of gambling beyond the individual, and focuses on external factors that may contribute to gambling‐related harm.^27^ Critical qualitative inquiry is important for public health research as it also considers the role of power, inequality, and injustice in contributing to health issues and advocating for social change.^28^, ^29^ Critical qualitative inquiries aim to expose inequality, place the voices of those impacted at the centre of inquiries, and seek to influence policy change.^26^, ^30^

### 2.2 Sampling and recruitment

To be eligible for the broader study, participants were required to identify as female, be aged 18 to 40 years, have gambled in the past 12 months, and speak a level of English that allowed them to confidently participate in the interview. This age range was chosen to incorporate experiences of both young and middle‐aged women, including those with and without children, women who had recently reached the legal age for gambling, and those who may have been gambling for longer periods of time.

Purposive and snowball techniques were used to recruit participants by promoting the study on social media sites, sending information to individuals who had taken part in prior studies and consented to be contacted for future research, and asking women to share information about the study via their networks. Those interested were sent a Plain Language Statement with information about the study and provided informed consent before participating in the interview. Prior to commencing, participants were reminded that the interview could be stopped or paused if there were any parts that made them feel uncomfortable. Participants were also directed to help services if they felt they needed to talk confidentially to someone about anything that arose from the interview. Women who participated received a $50 grocery voucher. Ethical approval was provided by Deakin University Human Research Ethics Committee (2019‐534).

### 2.3 Data collection

Semi‐structured interviews were conducted via telephone between July and December 2020. Interviews lasted approximately 1 hour and were audio‐recorded and subsequently transcribed by a professional transcription company and edited for accuracy by members of the research team. An interview guide was used to structure initial questions relating to participant socio‐demographics (age, state, relationship status, income), and risk of gambling harm. The nine‐item Problem Gambling Severity Index (PGSI) was used to measure level of gambling harm, whereby a score of 0 was classified as a nonproblem gambler, a score of 1‐2 was classified as a low‐risk gambler, a score of 3‐7 was classified as a moderate risk gambler, and a score of 8 or more was classified as a problem gambler.^31^ The broader study also collected data about personal gambling behaviours, normalisation of gambling in everyday environments, conceptualisation of risk behaviours and strategies to address the normalisation of gambling. The sections of the interview guide addressed in this paper related to women's reasons for engaging in gambling, and their perceptions of the risks associated with gambling. Examples of questions included: “*Why do you gamble?*”; “*What role does gambling play in your life?*”; and “*Do you think your own personal gambling has led you to take risks that you wouldn't normally have taken?*” For the broader study, data collection continued until interviews provided sufficient information to meet the aims of the project.

### 2.4 Data interpretation

The data were analysed using Braun and Clarke's^32^, ^33^ six phases of reflexive thematic analysis, with data specifically coded in relation to the research questions guiding this paper.^34^ ^(p34)^ This initially involved familiarisation with the data by listening to the audio‐recordings and re‐reading transcripts. The data were then coded and categorised using both open coding and focused coding techniques to identify and code data in alignment with the research aim and questions, and drawing upon theories of risk^23^, ^24^. This included exploring why young women gambled, their gambling practices, and their understanding of the risks and benefits of gambling. Initial codes included social gambling, aspirational gambling, gambling to relieve stress, the accessibility of gambling, the link between gambling to social and cultural institutions, family and peer influences and pressure, personal control and responsibility behaviours, and the benefits and risks of gambling. Ideas were developed in consultation with the research team. Initial themes were then generated by grouping codes with similar meanings, looking for patterns in the data, with the central concept for the potential theme identified by moving back and forth between the data and codes. Themes were reviewed and refined to ensure they reflected the research questions and demonstrated a pattern of shared meaning. The final stage of analysis involved defining themes and presenting the research findings as a narrative to explain factors that motivate young women's gambling. The research team met regularly to discuss and reflect on the interpretation of the data.

### 2.5 Participants

A total of 41 women, aged between 20 and 40 years (*M* = 30, SD: 6) participated in this study, and lived in Victoria (*n* = 24, 58.5%), New South Wales (*n* = 16, 39.0%) and South Australia (*n* = 1, 2.4%). Sixteen women (39.0%) were classified as low‐risk gamblers, eight (19.5%) were classified as moderate risk gamblers, and five (12.2%) were classified as problem gamblers. Twelve women were classified as non‐problem gamblers (29.3%).

## 3 RESULTS

Five themes were constructed from the data in relation to the research questions: (i) Gambling as a mechanism to escape everyday life; (ii) Gambling as a financial motivation to change life circumstances and opportunities; (iii) Gambling as a way of connecting with social networks; (iv) Gambling as an extension of leisure activities; and (v) Factors that lowered perceptions of the risks associated with gambling. Findings are presented below in relation to each theme.

### 3.1 Theme 1: Gambling as a mechanism to escape everyday life

Several women were motivated to gamble in order to escape aspects of their everyday life. These women predominantly gambled alone. For some women, gambling was perceived as a stress reliever, and they were motivated to gamble as a means of relaxation. These women often referred to the entertainment that gambling provided, and described seeking out gambling when they wanted “*something more exciting in the day*”. Some women who were mothers also described gambling at home when they had “*a quiet moment*” away from the kids. For example, one participant mentioned gambling on scratchies at home “*when the little one's sleeping*”. This reflected a broader pattern in which women described fitting gambling in with busy work and family schedules, including using gambling to take time out in their day.

Some women described going to gambling venues in the evening hours after work. The flexibility and accessibility of some gambling products appeared to facilitate these women's gambling behaviour:

*Usually I'll go to like a tavern or a pub. So, it's not like a hotel or a big club. Sometimes I'll go on my own or I'll go with my partner… It's either the weekend, but it's mostly after work. So, I would say like between, around that five to seven time. – 30‐year‐old, moderate risk gambler*.

For some women, gambling was perceived as an outlet and “*as a way of escaping*” the more difficult aspects of their lives. These participants commented that gambling environments were well suited to providing a break from life as they could go on their own and feel safe without having people speak to them. One woman stated that when she walked into a gambling venue, the outside world and her problems did not exist. Similarly, some women were motivated to gamble when they felt sad or lonely, and this included women who were classified as being at risk of gambling harm on the PGSI. These women often spoke about how their gambling filled a hole in their lives, and that they were motivated to gamble when they were feeling this way:

*I've always used gambling as a way of escaping…I always used to use venues as a way of being untouchable and nobody in there would – like I'd go in there for the purpose of not speaking to anyone, but just having a break from my life and the things that were going on around me. – 39‐year‐old, problem gambler*.

### 3.2 Theme 2: Gambling as a financial motivation to change life circumstances and opportunities

Some women who gambled alone were motivated by the possibility of winning significant amounts of money that could change their life. Typically, these women regularly gambled on lotteries or scratchies. They fantasised, “*what if I won that big jackpot or the big lotto?* ” and were motivated to gamble by the thought of becoming a millionaire. For example, one woman expressed how she continued to gamble in the hope of winning a large amount of money and that it was “*that hope that keeps you going*”. Another woman discussed how winning money through gambling could impact her life:

*If I win then that allows me to have some spending to do – whether it be pay my bills or buy something or, for me, all my family lives overseas, like winning the $10,000, I'm meant to be going to Malta this year… saving $10,000 with $50 a week it is going to take me bloody god knows how long. – 28‐year‐old, low‐risk gambler*.

Some women justified their engagement in gambling because they could easily fit the cost into their budget. For example, one woman rationalised her weekly lottery ticket by comparing it to the price of a cup of coffee. Another woman budgeted her regular visit to an EGM venue to ensure that she did not gamble more than intended, and that gambling would not impact on family expenses:

*That doesn't interfere into my family expenses so that doesn't create a problem… I'm sitting at a venue and gambling and betting and I lost. That eagerness comes into me that, "Oh, what, I lost, I need to get it back". But what stops me from doing that is my budget constraint. – 32‐year‐old, low‐risk gambler*.

### 3.3 Theme 3: Gambling as a way of connecting with social networks

Many women were motivated to gamble by the desire to connect with members of their social networks. For some participants, gambling was embedded in a broader social context *–* “*a social thing because it's always at a social event*”. For example, one woman described sharing a poker machine with her partner, while another incorporated gambling at a casino into date nights with her partner:

*I think most likely a Friday or a Saturday night when we want to have dinner in the casino. And then it's kind of like an add on after the dinner. So, we just pick some nice food and then afterwards, we go to the casino to have a bit of fun. – 33‐year‐old, low‐risk gambler*.

Some women commented that they were motivated to attend gambling venues over other social environments because they were accessible and open late. For example, one woman from a regional town stated that the local gambling venue was one of the few places in the area to socialise. Other women were motivated to visit the casino because it enabled them to prolong a night out with friends after the nearby bars and nightclubs had closed:

*It would probably be like a bar or a nightclub but then obviously now a lot of that closes quite early you know 1am, 2am, 3am, and you know some people still want to have another drink at the end of the night. So it's the only other place open and the only other real option is the casino. – 29‐year‐old, nonproblem gambler*.

As many social outings were linked with gambling environments, some women discussed feeling pressure to gamble or that gambling was integral to fitting in with their social network. This included one woman whose friends encouraged her to “*have a go*”, despite her not being interested. When asked what motivated her to gamble, she explained it was to fit in with the people she was socialising with. Another woman shared a similar experience:

*I think probably a combination of wanting to – like, not wanting to feel left out in my friendship group and boredom and I was like intrigued…– in fact, probably the most driving factor would be not wanting to be left out. – 22‐year‐old, low‐risk gambler*.

### 3.4 Theme 4: Gambling as an extension of existing leisure activities

Social outings with friends were sometimes located in gambling environments, and while gambling was not typically the purpose of such outings, women often engaged in gambling as an incidental activity. This was particularly the case for women who gambled on EGMs or casino table games because gambling venues facilitated social interactions:

*Usually there's a meal and some drinks involved. I mean, there definitely is. I don't really go to an establishment just to play the pokies. It's always because of a social situation where we will go and have a meal and drinks. – 34‐year‐old, nonproblem gambler*.

Other women who engaged in sports betting discussed the association between gambling and watching sport. For some, gambling increased the fun of watching sports because it added a personal investment in the outcome of the game. For example, some participants stated that gambling made watching sport “*more fun*” and “*more interesting"*, while others described being on the “*edge of their seats*” while watching sport because they had a stake in the game. Another woman described how gambling on events such as the Australian Football League grand final was common among her friends, and how these rituals led her to gamble on sports betting apps as well:

*…especially with the grand final sort of thing, it's what you do, the first goal you do and things like that, you just do it. It started where friends used to just sit there and you'd get a name out of a hat or whatever and you'd put a bit of money in and then you'd win as a group on grand final day. Then, moved on from doing that to doing it on an app instead. – 35‐year‐old, low‐risk gambler*.

Some women described being motivated to gamble on the sports they planned to watch. One woman commented that she was motivated to gamble after she would “*google the odds*” to find out who was the favourite to win a sporting match. Another stated that she would not bet on sporting events if it was not for her interest in sport. For others who were less interested in sport, gambling was a way of being involved with their partners' or friends' sporting interests. For example, some women indicated that they would not gamble on their own, but only when they watched sport with their partner:

*We like to do it together. But yeah, I wouldn't sit there and do it on my own or anything like that. – 37‐year‐old, low‐risk gambler*.

### 3.5 Theme 5: Factors that lowered perceptions of the risks associated with gambling

Many women who gambled perceived that their gambling was a low‐risk activity. There were several reasons for this. Some women who gambled on lotteries and scratchies reasoned that there were fewer risks associated with these products, with lotteries not really viewed as gambling. These products were perceived as being less addictive and as having a slower rate of play. For example, one woman described how the physical nature of purchasing scratchies in a store “*slow*[s] *you down*” compared to more accessible products. Others stated that it was the accessibility of gambling products which make them harmful:

*So, you think about like scratch tickets. You have to go in person to a physical shop to buy them. X amount of the time, it's going to take you a certain time to scratch through them, that's more than it's going to be. It's going to slow you down as opposed to playing pokies once a week, which is a hundred dollars at a time. And bet that all at the same time, you could run through a lot of money quite quickly. Or sports betting where you can do it just from your phone on an app while you're doing anything else. – 28‐year‐old, low‐risk gambler*.

*You could buy 50 scratch tickets a day and still have the same problem as a poker machine addict, but I think for me pokies are bad, because they're on every street corner, there's always signs out saying they're open when you're driving around. And I think that that is a really, really easy form of gambling to get addicted to. – 39‐year‐old, problem gambler*.

While many women recognised the risks of gambling for vulnerable groups and the broader community, some perceived that they were personally protected. These participants reasoned that gambling in social settings protected them from harm, while gambling alone increased the risk. For example, one woman suggested that attending the casino with her family prevented her from being able to gamble continuously and lose more than she intended:

*I think with my lifestyle, I wouldn't … I don't really go to the casino or anything like that, and if I do go to the casino or play on the pokies or something, I'm with my family. So, I don't think I'm in that opportunity where I could just sit there for a while or yeah, spend more money than I should. – 37‐year‐old, non‐problem gambler*.

The perception that gambling was not risky when done socially may have contributed to some women minimising the risks associated with their gambling. For example, one woman who was categorised as being a moderate risk gambler stated that she was “*not worried about*” the risks associated with her gambling. She described how she gambled with friends as “*something to do*” and implemented time and money limits:

*I feel like none of us are actually playing to win. We just like do the smallest bet. So, like one or two cent bets and we just did that for 15 minutes until it's all empty. And then leave. So, it's definitely more just like something to do. – 36‐year‐old, moderate risk gambler*.

When asked about the risk associated with their own gambling, it was common for women who were categorised as low‐ and moderate‐risk gamblers to state that there were no or limited risks. Many of these women referred to 'responsibility' measures that they felt protected them and kept them in control of their gambling. This included only gambling what they could afford to lose, setting time and money limits, only gambling for fun, and having a “*good sense*” of when to stop. One woman interpreted the control she had over her gambling as reducing the risks associated with her gambling:

*Facilitator: And are there any risks for you personally, do you think*?

*Participant: For me? No. I mean, I think there is, but I think we've got full control, so I'd say no. – 37‐year‐old, low‐risk gambler*.

Others compared their own gambling to those that they would consider as having a gambling addiction. For example, one woman indicated that her gambling was not risky as she had always been able to afford to put food on the table. Another suggested she was not at risk because she was aware of the risk of losing money, and she had not experienced withdrawal symptoms when the casino was closed in response to the COVID‐19 pandemic:

*No. I think I'm doing it in a responsible way…I do acknowledge the fact every time I go into a casino I am risking losing money. I do acknowledge that, I am aware of it. But I don't think it's at a risk. Because as I said before, I haven't gambled for a couple of months because of COVID, and it hasn't really…It's not like I'm getting a withdrawal to the point where I have to gamble online, as you said. So, I think personally I'm okay. – 25‐year‐old, moderate risk gambler*.

## 4 DISCUSSION

**FIGURE 1:**
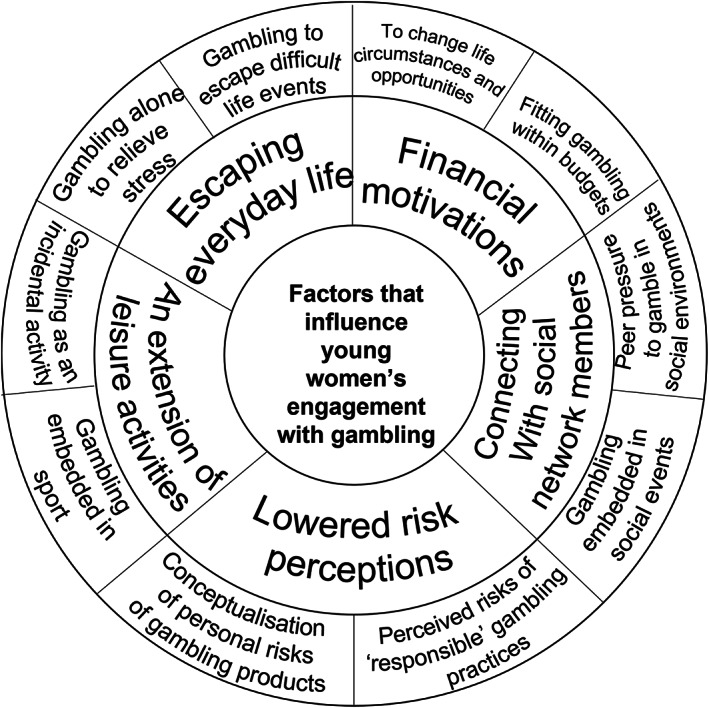
Factors that influence young women's engagement with gambling

The findings of this study suggest a range of factors that can influence young women's engagement with gambling and perceptions of associated risks. These factors may intertwine and produce environments that have significant appeal to women, and promote gambling as socially and culturally acceptable, and normalised activity (Figure 1).

Women's tendencies to use gambling to escape everyday life stressors has been previously identified in the literature.^7^ However, this study provides a more nuanced understanding about how different forms of gambling may play a role in providing 'time out' from everyday life, including work and carer responsibilities. Some women experienced this 'escape' by accessing different forms of gambling (such as scratchies) within their own homes. This finding is important for understanding how some women may experience a heightened risk of harms when participating in forms of gambling that are available and accessible at home. There are certain forms of gambling that traditionally appeal to women, such as lotteries, scratchies, bingo, and/or Keno, and are increasingly accessible online.^35^ The current study suggests that new forms of online gambling, such as betting on sport through apps, may also have increasing appeal to some women. They can be perceived as providing momentary escapes from everyday life, and hence may pose higher risks for younger women. It has been argued previously that the normalisation of gambling for younger people has partly occurred because of the expanded reach of online gambling products, the instant availability and accessibility of these products, and associated marketing strategies.^9^, ^36^, ^37^ However, most research and prevention activities that have addressed these issues have focused on young men.^36^, ^38^, ^39^ As different forms of gambling become normalised and socially acceptable for younger women, the overwhelming focus of harm prevention campaigns on young men's gambling^40^, ^41^, ^42^ will become difficult to justify.

Women acknowledged the risks of gambling to the broader community, however, some perceived that they were at low or no risk of experiencing gambling harm. Although traditionally gambling risk has been recognised as an individual determinant essentially requiring actions to inform ways to manage risk, this study reveals that younger women's perceptions of the risks associated with gambling are strongly influenced by socially *situational rationalities* ^24^ and *practical reasoning*.^43^ The present study indicates that women, as a networked social group, create gambling norms and rituals, which may vary across environments. This study demonstrates that such norms may reduce the capacity to recognise the risk (and protective) factors that are associated with gambling. For example, several conditions in the environment influenced the decisions of women to attend gambling venues, including the accessibility of these places late at night, their availability in regional areas, and the safety and social acceptability of spaces for women who were on their own. Some women in this study also reasoned that gambling in social groups afforded a level of protection because individuals would look out for each other. Other participants described acceptable and unacceptable ways of engaging with gambling. This included employing responsible gambling practices such as accounting for gambling within their household budgets, thus justifying that they could afford to lose a certain amount of money. These rationalisations were linked to women's perceived identities as mothers, carers and providers, and this suggests factors that influence women's engagement with gambling extend beyond recognised individual level factors and are situated in social and personal contexts. Recent initiatives have aimed to provide gamblers with personal responsibility guidelines about how to engage in 'safer' gambling.^44^ Suggestions include setting limits on the amount spent on gambling, limiting the frequency of gambling, and limiting the amount of products used to gamble.^44^ This study shows that a range of interconnected, complex individual, socio‐cultural and environmental factors contributed to women's conceptualisations of, and practical reasoning about gambling risk. In this context, the above reductionist guidelines may have little resonance with younger women, as they do not speak to the complexities that influence their risk perceptions and behaviours.

It should not be assumed that women's gambling practices and their engagement with different gambling products will remain unchanged over time. Research has demonstrated that gender has a critical influence on experiences of gambling, and it is important to further explore women's engagement with gambling products, their experiences of gambling harm, and the unique strategies to reduce or prevent gambling harm for different population subgroups of women.^5^, ^45^ Furthermore, understanding the gambling experiences of women will be important in challenging the harmful gender stereotypes and norms that are associated with women's gambling experiences. Public health initiatives should move away from personal responsibility paradigms and utilise specific evidence to inform education and information approaches that meet the needs of subgroups of women. This may involve providing accurate risk information about products and disrupting myths about gambling behaviours that may be perceived as a protective factor against gambling harm.

### 4.1 Limitations

The research is limited by the scope and coverage of the study sample. Efforts to recruit women aged 18 and 19 years were unsuccessful, and this study, therefore, does not represent the gambling behaviours and risk perceptions of those who may have recently started gambling legally. The study sample was restricted to women who spoke English. Future research is required to investigate the experiences of women from culturally and linguistically diverse backgrounds, and those from lower socioeconomic areas which may have higher concentrations of gambling venues. This research took a binary approach to gender which leaves out a more encompassing conceptualisation of gender and the notion of intersectionality. Finally, this study did not explore the commercial and political factors that are important in shaping women's perceptions of risk. This was beyond the scope of the study and should be considered for future research.

## 5 CONCLUSION

Public health and health promotion initiatives should recognise that women's gambling motivations and risk perceptions are diverse, and reflect a range of socio‐cultural and environmental factors that may influence younger women's engagement with gambling. These programs should be independent from the gambling industry, research‐based, adequately funded, and provide clear information about the risks of gambling products and environments. It is clear both that educational and information approaches aimed at preventing and reducing gambling harm in women must recognise the need for carefully researched and planned programs, and that these may in turn require nuance to meet the needs of specific target groups. Further, such programs must be part of a comprehensive public health approach that addresses the broader determinants of gambling harm.

## FUNDING INFORMATION

This study was funded by an ARC Discovery Grant (DP190100695). The funding body had no role in the design or write up of the study.

## CONFLICT OF INTEREST

The authors declare no conflict of interest but have a range of declarations. Simone McCarthy has received an Australian Government Research Training Program stipend from Deakin University for her PhD related to gambling and women. Samantha Thomas has received funding for gambling research from the Australian Research Council Discovery Grant Scheme, the Victorian Responsible Gambling Foundation, Healthway, and the New South Wales Office of Responsible Gambling. She has received travel expenses for gambling speaking engagements from the European Union, Beat the Odds Wales, the Office of Gaming and Racing ACT, SNSUS (Stiftelsen Nordiska Sällskapet för Upplysning om Spelberoende) and the Royal College of Psychiatry Wales. She is a member of the Responsible Gambling Advisory Board for LotteryWest. She does not receive any financial compensation for this role. Hannah Pitt has received funding for gambling research from the Australian Research Council Discovery Grant Scheme, VicHealth, Deakin University, the Victorian Responsible Gambling Foundation and the New South Wales Office of Responsible Gambling. Sarah Marko has received support for gambling research from an Australian Government Research Training Program Scholarship. Melanie Randle has received funding for gambling research from the Australian Research Council Discovery Grant Scheme and the Victorian Responsible Gambling Foundation. Sean Cowlishaw currently receives funding from the Australian Research Council and the Victorian Responsible Gambling Foundation for gambling‐related research. He has also received funding for mental health research from the National Health and Medical Research Council, the National Mental Health Commission, the Victorian Department of Health, the Victorian Department of Education & Training, The Teacher's Health Foundation, the State Trustees Australia Foundation, the Commonwealth Department of Veteran's Affairs and the Defence Health Foundation. Sean Cowlishaw has not knowingly received funding from the gambling industry or any industry sponsored organisation. He has participated in scholarly and policy related conferences and events which were sponsored by industry, but received no payment for involvement or expenses. Sylvia Kairouz has no competing financial interests to declare. She holds a Research Chair on Gambling funded by the Fonds de Recherche du Québec – Société et Culture (FRQ‐SC) and the Mise‐sur‐toi foundation. She received funding from the Canadian Institutes of Health Research (CIHR), the John Evans Leadership fund of the Canadian Foundation for Innovation, and the Social Sciences and Humanities Research Council of Canada (SSHRC). Mike Daube has received funding for gambling research from the Australian Research Council Discovery Grant Scheme and the Victorian Responsible Gambling Foundation Grants Scheme.
